# Patients’ characteristics and 30-day mortality for those undergoing elective surgeries during the COVID-19 pandemic in Bangladesh

**DOI:** 10.1371/journal.pone.0289878

**Published:** 2023-08-14

**Authors:** Shakera Ahmed, Anwarul Karim, Tanvir Kabir Chowdhury, Orindom Shing Pulock, Nowrin Tamanna, Mastura Akter, Puja Biswas, Fahmida Afroz, Susmita Dey Pinky, Anika Nahrin Alabbi, Tasnuba Raisa Jamil, Zarin Tasnim, Dipa Dev, Mraching Marma, Tasmiah Tahera Aziz, Hafiz Ahmed Nazmul Hakim, A. K. M. Khairul Basher, Nur Hossain Bhuiyan Shahin, Tahmina Banu

**Affiliations:** 1 Chittagong Research Institute for Children Surgery, Chattogram, Bangladesh; 2 Chittagong Medical College and Hospital, Chattogram, Bangladesh; 3 School of Clinical Medicine, Li Ka Shing Faculty of Medicine, The University of Hong Kong, Hong Kong, Hong Kong SAR; 4 University of South Carolina, Columbia, SC, United States of America; 5 Department of Biomedical Sciences, City University of Hong Kong, Hong Kong, Hong Kong SAR; 6 Dhaka Medical College and Hospital, Dhaka, Bangladesh; North South University, BANGLADESH

## Abstract

**Background:**

The COVID-19 pandemic has significantly impacted the surgical practice throughout the world, including elective surgical care. This study investigated the characteristics of patients undergoing elective surgery, the prevalence of COVID-19 infection, the surgical procedures performed, and 30-day mortality in general and pediatric surgical settings in selected tertiary-level hospitals in Bangladesh from November 2020 to August 2021.

**Methods:**

This serial cross-sectional study included 264 patients scheduled for elective surgeries during the study period. All patients underwent COVID-19 real-time polymerase chain reaction (RT-PCR) testing within 24 hours before surgery. Data on age, sex, common comorbidities, surgical procedures, and 30-day mortality were collected and analyzed. Furthermore, comparisons were made between COVID-19 positive and negative patients.

**Results:**

The prevalence of COVID-19 infection among patients was 10.6%. Older age, a history of major surgery within the last three months, hypertension, and diabetes mellitus were significantly associated with COVID-19 infection. All COVID-19-negative patients underwent surgery, while only 46.4% of COVID-19-positive patients underwent surgery. The most common surgical procedures were related to the digestive system, breast, and urinary system. Only one patient (0.4%) died within 30 days after surgery among the COVID-19-negative patients, whereas two patients (7.1%) died among the COVID-19-positive patients: one before surgery and one after surgery.

**Conclusions:**

This study provides valuable insights into the characteristics, burden of COVID-19 infection, and 30-day mortality of patients undergoing elective surgery in tertiary care centers in Bangladesh during the pandemic.

## Introduction

The COVID-19 pandemic has had a significant impact on almost every aspect of healthcare delivery and surgical care [1–3]. Patients requiring surgical management are at high risk of exposure to severe acute respiratory syndrome coronavirus 2 (SARS-CoV-2), both in community settings and at hospitals. COVID-19 has been shown to be an independent risk factor for surgical mortality [4]. During the peak months of the COVID-19 pandemic, mortality rates among surgical patients increased, regardless of their COVID-19 status [5]. A number of guidelines [6], including those from Bangladesh [7], were published during the pandemic’s initial stages for managing surgical patients. The majority of these guidelines recommended postponing elective surgeries while still performing emergency and urgent surgeries regardless of COVID-19 infection status.

The pandemic has had a significant impact on the provision of elective surgeries worldwide, with estimates suggesting that up to 72% of elective surgeries have been canceled or postponed, leading to an overwhelming elective surgical backlog [8]. A study conducted during the COVID-19 pandemic predicted that, in England and Wales, at least 2.4 million surgical procedures would have been canceled by the end of 2021 [9]. Similarly, Brazil’s elective surgical backlog surpassed 900,000 cases by December 2020 [10]. In Italy, a remarkable reduction of activity in surgical oncology resulted in a doubled waiting list [11]. In the United States, there was a dramatic change in surgical practices during the pandemic, with rapidly decreasing numbers of elective surgeries as a result of efforts to decrease disease transmission, conserve personal protective equipment, and widespread recommendations [12]. Despite the fact that the surgical provision for these patients is elective, delaying even benign surgeries can also have a significant impact on patients’ health, causing discomfort, decreased quality of life, and depression [13].

As the pandemic is transitioning into an endemic phase, healthcare systems worldwide are updating strategies for providing surgical care and clearing the backlog of elective surgeries, which could take years. Multiple organizations have already provided guidelines for resuming elective surgeries [13]. In this context, it is imperative to investigate and document the characteristics and outcomes of patients undergoing elective surgery in different healthcare settings and countries. This will help us better understand the dynamic impact of the pandemic on elective surgical care and inform evidence-based clinical practice and public health policies. Although studies from different countries investigating such issues in this drastically changed landscape have been published [5, 14–16], there is a scarcity of similar studies from Bangladesh in the literature, especially in relation to elective surgery. Due to the differences in healthcare systems as well as socioeconomic contexts among different countries, data from other countries may not represent the context of another country, including Bangladesh. Moreover, although studies from other populations have shown an association between various factors, such as hypertension, diabetes mellitus, hospital stays, and smoking history with the risk of COVID-19, such data about Bangladeshi population is scarce [17–20]. Therefore, this study aims to investigate the characteristics of patients undergoing elective surgery, the prevalence of COVID-19 infection and other common comorbidities among them, the surgical procedures performed, and mortality within 30 days of surgery in general and pediatric surgical settings in selected tertiary-level hospitals in Bangladesh from November 2020 to August 2021. This information could be useful in assessing the characteristics of patients and the impact of the COVID-19 pandemic on those undergoing elective surgery. Additionally, it could also aid in informing guidelines for the management of surgical patients during such pandemic disruptions, especially in Bangladesh.

## Methods

This serial cross-sectional study was conducted from November 2020 to August 2021. Data were collected from the Department of Surgery and Department of Pediatric Surgery of Chittagong Medical College Hospital, the Department of Surgery of Dhaka Medical College Hospital, and Dhaka Shishu Hospital. Ethical clearance was obtained from the ethical review committee of Chittagong Medical College, Chattogram, Bangladesh. We employed a convenience sampling method, patients who were planned for elective surgical procedures during the study period were prospectively recruited in this study, while emergency surgical cases were excluded. Informed written consent was taken from all patients. Due to the unpredictable nature of the COVID-19 pandemic and the associated logistical challenges during the study period, a predefined sample size was not calculated. Instead, we recruited as many eligible patients as possible during the study period. All patients included in this study underwent a COVID-19 real-time polymerase chain reaction (RT-PCR) test within 24 hours prior to surgery. The surgical procedures performed were categorized under the Current Procedural Terminology (CPT®) coding system. The outcome measure of this study was mortality within 30 days of the primary surgical procedure. Furthermore, in addition to the age and sex of the patients, other important and easily obtainable characteristics and comorbidities from medical documents and patients’ history were recorded. These included major surgery within the last three months, hypertension, diabetes mellitus, smoking history, American Society of Anesthesiologists (ASA) class, anesthesia used, and the primary surgeon’s rank.

Data were collected at each center using a preformed data collection sheet, and the analysis was conducted using IBM SPSS Statistics version 26. Both descriptive and inferential statistics were used in the results. Categorical data were expressed as frequency and percentage, and the Chi-square test was used to compare them. Continuous data were expressed as mean and standard deviation, and the Mann-Whitney Test was used to test the difference between groups. A *p* value of <0.05 was considered statistically significant.

## Results

In this study, 264 patients (n = 264) were included, with a mean age of 35.2 ± 20.5 years (range: 2 days to 75 years) (Table 1). Among them, 126 (47.7%) were male, and 138 (52.3%) were female. Thirty-two (12.1%) patients had undergone major surgery within the last three months. Hypertension and diabetes mellitus were present in 21 (8%) and 24 (9.1%) patients, respectively. A smoking history (current or previous smoking for at least one year regardless of pack-years) was present in 53 (20.1%) patients.

**Table 1:** Demographic and preoperative characteristics of patients.

Variable		Total patients n = 264	COVID-19 (-) n = 236 (89.4%)	COVID-19 (+) n = 28 (10.6%)	*p* value
Age, years (mean ± SD)		35.2 ± 20.5	33.9 ± 20.7	45 ± 15.1	.006
Age groups					
	<10 years	39 (14.8%)	39 (16.5%)		
	10–19 years	27 (10.2%)	26 (11.0%)	1 (3.6%)	
	20–29 years	38 (14.4%)	34 (14.4%)	4 (14.3%)	
	30–39 years	41 (15.5%)	38 (16.1%)	3 (10.7%)	
	40–49 years	36 (13.6%)	27 (11.4%)	9 (32.1%)	
	50–59 years	41 (15.5%)	37 (15.7%)	4 (14.3%)	
	60–69 years	34 (12.9%)	28 (11.9%)	6 (21.4%)	
	≥ 70 years	8 (3.0%)	7 (3.0%)	1 (3.6%)	
Sex					
	Female	138 (52.3%)	124 (52.5%)	14 (50%)	.843
	Male	126 (47.7%)	112 (47.5%)	14 (50%)	
Major surgery in last three months					
	Yes	32 (12.1%)	24 (10.2%)	8 (28.6%)	.010
	No	232 (87.9%)	212 (89.8%)	20 (71.7%)	
Hypertension					
	Yes	21 (8.0%)	12 (5.1%)	9 (32.1%)	< .001
	No	243 (92.0%)	224 (94.9%)	19 (67.9%)	
Diabetes mellitus					
	Yes	24 (9.1%)	16 (6.8%)	8 (28.6%)	.001
	No	240 (90.9%)	220 (93.2%)	20 (71.4%)	
Smoking history					
	Yes	53 (20.1%)	49 (20.8%)	4 (14.3%)	.617
	No	211 (79.9%)	187 (79.2%)	24 (85.7%)	

### COVID-19 infection and associated factors

The prevalence of COVID-19 infection among the patients included in this study was 10.6% (28/264). As shown in Table 1, COVID-19 positive patients (age 45 ± 15.1 years) were comparatively older than COVID-19 negative patients (age 33.9 ± 20.7 years), with *p* = 0.006. No significant difference in sex distribution was observed between COVID-19-positive and negative patients (*p* = 0.84). Among COVID-19-positive patients, 28.6% (8/28) had a history of major surgery in the last three months, whereas it was only 10.2% (24/236) in COVID-19-negative patients (*p* = 0.01). Both hypertension and diabetes mellitus were present in significantly higher proportions (32.1% and 28.6%, respectively) among patients with COVID-19 infection compared to patients without COVID-19 (5.1% and 6.8%), with *p* < .001 and *p* = 0.001, respectively. However, smoking history was not found to be associated with COVID-19.

To further investigate the relationship between these variables and COVID-19 positivity, a multiple regression analysis was conducted using the stepwise method (Table 2). The analysis showed that hypertension (B = .355, *p* < .001) and history of major surgery in the last three months (B = .171, *p* = .002) were both significantly associated with COVID-19 infection (R^2^ = .128).

**Table 2:** Multiple regression analysis predicting COVID-19 infection.

Model	B	SE	t	*p* value	95% Confidence Interval for B
(Constant)	1.057	.020	53.549	< .001	1.018–1.096
Hypertension	.355	.066	5.397	< .001	.226 - .485
Major surgery in last three months	.171	.055	3.128	.002	.063 - .278

### ASA class, type of anesthesia used, and surgeon’s rank for patients undergoing surgery

All COVID-19 negative patients (n = 236) underwent their planned elective surgery. However, among the COVID-19 positive patients (n = 28), only 13 (46.4%) patients underwent surgery. The surgery was postponed for the remaining 15 (53.6%) COVID-19 positive patients. The decision of whether to perform or postpone the surgery was based on multiple factors, such as the urgency of surgical management, severity of COVID-19 symptoms, and the need for referral to dedicated COVID-19 wards. However, detailed documentation of the factors justifying such decisions for each patient was not done in this study.

Table 3 displays the ASA class, type of anesthesia used, and the primary surgeon’s rank for the patients who underwent surgery (n = 249). The majority (180/249 = 72.3%) of the patients belonged to ASA I or II. Most patients (207/249 = 83.1%) received general anesthesia during surgery. Assistant professors served as the primary surgeon in nearly half of the surgeries (119/249 = 47.8%), while the remaining surgeries were performed by junior surgeons, associate professors, and professors.

**Table 3:** Characteristics of patients who underwent surgery.

Variable		Total patients n = 249	COVID-19 (-) n = 236 (94.8%)	COVID-19 (+) n = 13 (5.2%)	*p* value
Age, years (mean ± SD)		34.64 ± 20.68	33.94 ± 20.71	47.15 ± 15.88	.025
Age groups					
	<10 years	39 (15.7%)	39 (16.5%)		
	10–19 years	27 (10.8%)	26 (11%)	1 (7.7%)	
	20–29 years	35 (14.1%)	34 (14.4%)	1 (7.7%)	
	30–39 years	39 (15.7%)	38 (16.1%)	1 (7.7%)	
	40–49 years	31 (12.4%)	27 (11.4%)	4 (30.8%)	
	50–59 years	39 (15.7%)	37 (15.7%)	2 (15.4%)	
	60–69 years	32 (12.9%)	28 (11.9%)	4 (30.8%)	
	≥ 70 years	7 (2.8%)	7 (3%)		
Sex					
	Female	129 (51.8%)	124 (52.5%)	5 (38.5%)	.398
	Male	120 (48.2%)	112 (47.5%)	8 (61.5%)	
Major surgery in last three months					
	Yes	29 (11.6%)	24 (10.2%)	5 (38.5%)	.010
	No	220 (88.4%)	212 (89.8%)	8 (61.5%)	
Hypertension					
	Yes	16 (6.4%)	12 (5.1%)	4 (30.8%)	.006
	No	233 (93.6%)	224 (94.9%)	9 (69.2%)	
Diabetes mellitus					
	Yes	19 (7.6%)	16 (6.8%)	3 (23.1%)	0.066
	No	230 (92.4%)	220 (93.2%)	10 (76.9%)	
Smoking history					
	Yes	52 (20.9%)	49 (20.8%)	3 (23.1%)	.737
	No	197 (79.1%)	187 (79.2%)	10 (76.9%)	
ASA class					
	I	133 (53.4%)	125 (53.0%)	8 (61.5%)	
	II	47 (18.9%)	44 (18.6%)	3 (23.1%)	
	III	13 (5.2%)	13 (5.5%)		
	IV	35 (14.1%)	33 (14.0%)	2 (15.4%)	
	V	21 (8.4%)	21 (8.9%)		
	I and II combined	180 (72.3%)	169 (71.6%)	11 (84.6%)	.307
	III, IV, and V combined	69 (27.7%)	67 (28.4%)	2 (15.4%)	
Anesthesia given					
	General	207 (83.1%)	195 (82.6%)	12 (92.3%)	.364
	Spinal / Local	36 (14.5%) / 6 (2.4%)	35 (14.8%) / 6 (2.5%)	1 (7.7%) / 0	
Rank of the primary surgeon					
	Resident/ Medical officer/ Registrar	42 (16.9%)	38 (16.1%)	4 (30.8%)	.353
	Assistant professor	119 (47.8%)	112 (47.5%)	7 (53.8%)	
	Associate professor	53 (21.3%)	52 (22.0%)	1 (7.7%)	
	Professor	35 (14.1%)	34 (14.4%)	1 (7.7%)	
30-day mortality					
	No	247 (99.2%)	235 (99.6%)	12 (92.3%)	.102
	Yes	2 (0.8%)	1 (0.4)	1 (7.7%)	

### Surgical procedures performed and mortality rates

Surgical procedures were categorized according to the CPT coding system. As shown in Table 4, the majority of patients underwent surgical procedures related to the digestive system (n = 163, 65.5%), followed by procedures on the breast (n = 59, 23.7%) and urinary system (n = 15, 6%).

**Table 4:** Surgical procedures categorized by CPT coding.

Procedure (CPT code)			Total patients n = 249	COVID-19 (-) n = 236 (94.8%)	COVID-19 (+) n = 13 (5.2%)
Integumentary System (10030–19499)			62 (24.9%)	58 (24.6%)	4 (30.8%)
	Incision and Drainage Procedures on the Skin, Subcutaneous and Accessory Structures		3 (1.2%)	3 (1.3%)	
	Surgical Procedures on the Breast		59 (23.7%)	59 (25.0%)	
		Aspiration, Incision and Drainage Procedures of Breast	3 (1.2%)	3 (1.3%)	
		Ablation, Exploration and Excision Procedures	26 (10.4%)	26 (11.0%)	
		Mastectomy Procedures	30 (12.0%)	26 (11.0%)	4 (30.8%)
Respiratory System (30000–32999)			3 (1.2%)	3 (1.3%)	
	Surgical Procedures on the Lungs and Pleura		3 (1.2%)	3 (1.3%)	
Hemic and Lymphatic Systems (38100–38999)			4 (1.6%)	4 (1.7%)	
	Excision Procedures on the Spleen		3 (1.2%)	3 (1.3%)	
	Excision Procedures on the Lymph Nodes and Lymphatic Channels		1 (0.4%)	1 (0.4%)	
Digestive System (40490–49999)			163 (65.5%)	154 (65.3%)	9 (69.2%)
	Incision Procedures on the Tongue and Floor of Mouth		1 (0.4%)	1 (0.4%)	
	Excision Procedures on the Esophagus		1 (0.4%)	1 (0.4%)	
	Surgical Procedures on the Stomach		14 (5.6%)	11 (4.7%)	3 (23.1%)
	Surgical Procedures on the Intestines (Except Rectum)		42 (16.9%)	39 (16.5%)	3 (23.1%)
	Surgical Procedures on the Appendix		5 (2.0%)	5 (2.1%)	
	Excision Procedures on the Rectum		9 (3.6%)	9 (3.8%)	
	Surgical Procedures on the Anus		11 (4.4%)	11 (4.7%)	
	Surgical Procedures on the Biliary Tract		31 (12.4%)	30 (12.7%)	1 (7.7%)
	Surgical Procedures on the Pancreas		3 (1.2%)	3 (1.3%)	
	Surgical Procedures on the Abdomen, Peritoneum, and Omentum		46 (18.5%)	44 (18.6%)	2 (15.4%)
		Incision Procedures on the Abdomen, Peritoneum, and Omentum	28 (11.2%)	27 (11.4%)	1 (7.7%)
		Laparoscopic Procedures on the Abdomen, Peritoneum, and Omentum	1 (0.4%)	1 (0.4%)	
		Hernioplasty, Herniorrhaphy, Herniotomy Procedures	17 (6.8%)	16 (6.8%)	1 (7.7%)
Surgical Procedures on the Urinary System (50010–53899)			15 (6.0%)	15 (6.4%)	
Surgical Procedures on the Male Genital System (54500–54699)			5 (2.0%)	5 (2.1%)	
Surgical Procedures on the Female Genital System (56405–58999)			2 (0.8%)	2 (0.8%)	

Among the COVID-19 negative patients (n = 236), one patient with esophageal carcinoma who underwent feeding jejunostomy tube placement died on the 4^th^ postoperative day (mortality rate 0.4%) (Table 5). Among the COVID-19 positive patients, one patient died due to severe COVID-19 pneumonia before surgery, and another patient with intestinal obstruction from advanced rectal carcinoma, who underwent ileostomy surgery, died from severe COVID-19 pneumonia on 3^rd^ postoperative day.

**Table 5:** Description of patients who died within 30 days of surgery.

Patient	Age (years)	Sex	COVID-19 status	Primary Dx	Types of surgery	Cause of death	Interval from surgery
1	60	Male	Negative	Carcinoma of esophagus	Feeding jejunostomy	Sepsis	4^th^ POD
2	48	Female	Positive	Intestinal obstruction due to advanced carcinoma of rectum	Trephine loop ileostomy	COVID-19 pneumonia	3^rd^ POD
3	30	Male	Positive	Choledocholithiasis	N/A	COVID-19 pneumonia	N/A

N/A: Not applicable; the patient died before undergoing surgery.

## Discussion

The COVID-19 pandemic has significantly disrupted healthcare systems worldwide, presenting unprecedented challenges and obstacles [1–3, 21–23]. Bangladesh announced its first case of COVID-19 on March 8, 2020 [24]. As one of the most densely populated countries in the world with limited healthcare resources, the country has faced immense pressure on its healthcare system since the beginning of the pandemic. During the initial stages of the COVID-19 outbreak, Bangladesh, like many countries, followed recommendations for managing elective surgical cases and postponed a significant portion of such procedures to minimize the risk of COVID-19 transmission among patients and healthcare providers, to reduce the risk of unfavorable outcomes after surgery, and to address the unprecedented healthcare crisis resulting from the surge in COVID-19 cases. Although several studies from different countries have reported on the characteristics and outcomes of patients undergoing elective surgery during the pandemic, there is a scarcity of such data from Bangladesh. The present study aimed to examine the characteristics, burden of COVID-19, and 30-day mortality of patients undergoing elective surgeries in tertiary care centers during the pandemic in Bangladesh.

The present study found that the prevalence of COVID-19 infection among patients planned for elective surgery was 10.6%. This figure is lower than the national data from the Directorate General of Health Services (http://dashboard.dghs.gov.bd/webportal/pages/covid19.php), which revealed a laboratory confirmed case rate of 20.3% among the 1,092,934 laboratory tests conducted during the study period. The lower rate of COVID-19 cases in our study cohort can be attributed to the mandatory laboratory testing conducted for all included patients, irrespective of their signs, symptoms, or contact history with COVID-19 patients. In contrast, the national data may have been influenced by a larger proportion of laboratory tests conducted on individuals who were suspected of having COVID-19 due to their signs, symptoms, or contact history. Additionally, our study’s patient population primarily consisted of elective surgery cases, which are generally considered for individuals presenting with minimal or no symptoms. More symptomatic cases are likely to be transferred to designated wards or placed under quarantine before being seen by the surgical wards included in this study if their surgical treatment could be postponed. As a result, this may have contributed to a lower proportion of COVID-19 cases in our cohort.

In this study, older age, a history of major surgery within the last three months, hypertension, and diabetes mellitus were found to be significantly associated with COVID-19 infection among elective surgical cases. Multiple regression analysis revealed that history of major surgery within the last three months and hypertension could explain 12.8% of the variance in COVID-19 test results in our patient cohort. Older age is a known risk factor for COVID-19 infection, as well as for a higher risk of severe COVID-19 disease [25, 26]. Multiple studies have reported hypertension and diabetes mellitus as common comorbidities in patients with COVID-19 [17, 18]. The association between COVID-19 and a higher rate of history of recent surgery is in line with previous reports of increased COVID-19 risk in hospitalized patients due to hospital-acquired infections [19, 20]. Age and other related risk factors, such as hypertension and diabetes mellitus, often coexist and may increase the risk of contracting COVID-19 and subsequent development of severe disease [26–28].

Current study found no significant difference in the sex distribution between COVID-19 positive and negative patients. Although different studies have reported varying ranges of sex distribution among COVID-19 positive patients, a systematic review and meta-analysis showed that the pooled prevalence of COVID-19 confirmed cases was higher in men at 55.00 (51.43–56.58, I^2^ = 99.5%, p<0.001) compared to women at 45.00 (41.42–48.57) [29]. This indicates that COVID-19 is more prevalent in men than in women. The difference between this meta-analysis and our study could be due to the smaller number of COVID-19 positive patients in our study.

In our study, we found a positive smoking history of 20.8% and 14.3% among COVID-19 negative and positive patients, respectively, who underwent elective surgery. Although reports on the prevalence of smoking history among patients undergoing elective surgery during both the pre-pandemic and pandemic eras are scarce in Bangladesh, our current study’s findings are consistent with the prevalence of smoking history (20%) reported from Pakistan [30]. While many studies have reported a significant association between smoking and COVID-19 [31–33], other studies have reported an unusually low prevalence of smoking among patients [34, 35]. The observed heterogeneity of the association between smoking and COVID-19 could be due to differences in sample size, study population, study design, incomplete data reporting, as well as differences in defining the "smoking" history.

A wide range of mortality rates has been reported in patients undergoing surgery during the pandemic in various studies. These studies differ significantly in study design, timing of the study during the pandemic, sample size and characteristics, type of surgery and urgency of procedures, duration of follow up, as well as the standard of care. Therefore, direct comparison between studies is challenging. While zero mortality was reported in a study of 494 cases of elective major cancer surgery [36], other studies, particularly those that included emergency surgical cases, reported significantly higher mortality rates [5, 8, 14, 16]. In our study, although higher mortality was observed among COVID-19 positive patients compared to COVID-19 negative patients consistent with other studies [37], the overall mortality in both groups was on the lower end of the spectrum. However, substantially larger studies incorporating patients from various departments across multiple centers are necessary to enable better estimation of mortality rates among elective surgical patients, depending on their ASA classes and disease severity.

During this pandemic period, the most frequently performed surgeries involved the digestive system, followed by the integumentary system (mainly surgical procedures on the breast), and the urinary system. This is consistent with reports from the United States [38] which showed that even during the surge in COVID-19 cases, resulting in unprecedented disruptions to hospital operations, surgery was most frequently performed on gastrointestinal oncology patients and patients with acute gastrointestinal bleeding for whom alternative therapy was not appropriate.

Moreover, our study sheds light on the characteristics of patients undergoing elective surgery by reporting the ASA class, rank of the surgeons, and the type of anesthesia used. The majority of patients who underwent surgery belonged to ASA class I or II, indicating a generally lower burden of other systemic illnesses among these patients. However, comparable data from the pre-pandemic era in Bangladesh is scarce. Assistant professors, who typically hold specialist qualifications after completing several years of specialty training and subsequently have accumulated few years of experience in providing direct clinical service in an academic hospital, acted as the primary surgeons in the majority of elective surgeries conducted during the study period. This highlights their significant role as major surgical care providers during the pandemic.

Our study faced several limitations, such as being conducted in selected departments of a few tertiary care centers in Bangladesh, which might limit its generalizability to other settings or countries. The sample size was relatively small, particularly for COVID-19 positive patients, potentially affecting the statistical power. Focusing on elective surgical patients restricts the applicability to other patient groups, such as those requiring emergency surgery or medical care. Aside from the characteristics investigated in the study, other comorbidities, such as preexisting cardio-pulmonary diseases, immunosuppressive diseases, or other chronic systemic diseases, which are similarly important, were not included in the study. Furthermore, long-term outcomes or other complications of surgery apart from the 30-day mortality rate were not included. This was due to the logistical challenges of conducting such a study in the existing pandemic burden and manpower crisis within the already resource-limited healthcare setting. Despite its limitations, our study provides valuable insights into the characteristics and mortality of patients undergoing elective surgery in general and pediatric surgical settings at selected tertiary-level hospitals in Bangladesh during the study period. Our findings could serve as a foundation for further large-scale investigations or contribute to meta-analyses of similar studies to obtain more robust results that could be used to inform clinical practice and policies.

## Conclusion

In conclusion, this study provides valuable insights into the characteristics, burden of COVID-19 infection, and 30-day mortality of patients undergoing elective surgery in tertiary care centers in Bangladesh during the pandemic. The prevalence of COVID-19 infection among elective surgical patients was found to be 10.6%, with older age, a history of major surgery within the last three months, hypertension, and diabetes mellitus being significantly associated with COVID-19 infection. Most patients in our study group received surgery related to the digestive system. The overall mortality rate among our patients was relatively low, with higher mortality observed among COVID-19 positive patients compared to COVID-19 negative patients.

## Supporting information

S1 File Inclusivity in global research. (DOCX)
